# The Myometric Assessment of Achilles Tendon and Soleus Muscle Stiffness before and after a Standardized Exercise Test in Elite Female Volleyball and Handball Athletes—A Quasi-Experimental Study

**DOI:** 10.3390/jcm13113243

**Published:** 2024-05-31

**Authors:** Claudia Römer, Julia Czupajllo, Bernd Wolfarth, Freddy Sichting, Kirsten Legerlotz

**Affiliations:** 1 Department of Sports Medicine, Charité University Medicine Berlin, 10115 Berlin, Germany; 2 Department of Human Movement Science, Chemnitz University of Technology, 09111 Chemnitz, Germany; 3 Movement Biomechanics, Institute of Sport Sciences, Humboldt University Berlin, 10115 Berlin, Germany

**Keywords:** myometry, tendon, muscle, professional female athlete, type of sport

## Abstract

**Background:** The high prevalence of injuries in female athletes necessitates a course of action that not only enhances research in this field but also incorporates improved prevention programs and regular health monitoring of highly stressed structures such as tendons and muscles. Since myometry is already used by coaches and physiotherapists, it is important to investigate whether tissue stiffness varies in different types of sports, and whether such measures are affected by an acute training session. **Methods:** Myometric measurements of the Achilles tendon (AT) and soleus muscle (SM) were performed in the longitudinal plane and relaxed tendon position. In total, 38 healthy professional female athletes were examined, applying a quasi-experimental study design, with subgroup analysis performed for different sports. To investigate the stiffness of the AT and SM, 24 female handball and volleyball athletes performed a standardized maximal incremental performance test on a treadmill. In this subgroup, myometric measurements were taken before and after the exercise test. **Results:** The measurements showed no significant difference between the mean pre- (AT: 661.46 N/m; SM 441.48 N/m) and post-exercise stiffness (AT: 644.71 N/m; SM: 439.07 N/m). Subgroup analysis for different types of sports showed significantly lower AT and SM stiffness in swimming athletes compared to handball (*p* = 0.002), volleyball (*p* = 0.000) and hammer throw athletes (*p* = 0.008). **Conclusions:** Myometry can be performed on the same day as an acute training session in healthy female professional volleyball and handball athletes. Female swimmers have significantly lower AT and SM stiffness compared to female handball, volleyball and hammer throw athletes. These results show that the stiffness differences in the AT and SM can be assessed by myometry.

## 1. Introduction

In 2024, there will be parity between women and men at the Olympic Games in Paris for the first time [1]. Despite this outstanding development in elite sport, women in competitive sports continue to face major challenges at the youth and adult ages due to a less well-developed health infrastructure. Disparities in funding and resources for professional female athletes not only lead to a gender data gap in research but also result in less access to high-quality sports medical care, diagnostics and strength and stability coaches. Furthermore, a lack of data on injury prevention programs among women in competitive sports still exists [2]. Specialists in sports medicine, as well as menstrual health management and mental health specialists, are necessary in a female athlete’s career to address early issues such as pre-menstrual syndrome or pregnancy-related concerns. Poorly developed prevention structures in professional female sports and lower remuneration often lead to rudimentary medical and physiotherapeutic care compared to male competitive athletes. Myometry is already used in competitive and recreational sports to measure differences in stiffness in various musculoskeletal tissues [3,4,5], and it may help close this data gap.

### 1.1. Myometry Application and Measurement

The handheld device for myometry, MyotonPro (MyotonPro^®^, Myoton AS, Tallin, Estonia), is easy to use and can be operated by non-medical personnel. It measures the oscillation response of musculoskeletal tissue via a standardized external impulse (force 0.4 N).

The integrated accelerometer measures the oscillation and displays the oscillation curve and stiffness (N/m) on the screen [6]. The correct contact pressure is indicated to the examiner by means of color-coded light. The measurement can be performed quickly and in the same standardized examination position at the examination site or in physiotherapy.

### 1.2. Myometry in the General Population

Myometry demonstrates good intra- and inter-rater reliability for muscles and tendons [7,8,9,10,11,12]. Data indicate a good between-day reliability for the lower limb in healthy elderly men [7] and a moderate to high reliability for passive and active contractions [12]. Specifically for the Achilles tendon (AT), there is high intra- and inter-rater reliability [5,13]. Furthermore, myometry shows significant differences in AT stiffness at different angles of dorsiflexion [14]. Like other stiffness assessment techniques, such as shear wave elastography, myometry can also detect stiffness differences in the AT and SM in different ankle positions [15,16], and it shows significantly higher stiffness in voluntarily contracted muscles compared to those at rest in healthy individuals [11].

Using myometry, age-related correlations were identified by Gervasi et al. [17]. Older sedentary controls exhibited higher values of dynamic stiffness (N/m) compared to active controls [17]. In an identical twin study, myometry detected significantly higher Achilles tendon stiffness in the recreationally active twin compared to the inactive one [18]. It was also shown that AT stiffness adaptation could be specific to the sport type [18] due to different locomotion patterns.

### 1.3. Myometry in Sports

Myometry can be applied in sports to detect significant stiffness differences in the musculoskeletal tissue of symptomatic athletes, which can be indicative of tendinopathy [19,20]. Male athletes have been included more frequently in recent studies [4,21,22,23,24] and myometry data for professional female athletes in different type of sports are still lacking. Pruyn et al. conducted a study to assess muscle and tendon stiffness using myometry in female athletes at different competition levels [25]. Elite team sport players exhibited higher active stiffness compared to sub-elite players. These findings provide important insights into gender-specific stiffness and further research is necessary to evaluate myometry for more regular use in female sports. Pruyn et al. examined 15 female netball players and showed the good in-between-day reliability of myometry for athletes who avoided training sessions 24 h before measurement [26]. Data before and after acute exercise for professional female athletes, which could evaluate whether a single training session causes differences in dynamic stiffness measured by myometry, remain unavailable. The primary aim of this study is to examine the dynamic stiffness (N/m) of the Achilles tendon and soleus muscle in healthy female professional athletes before and after a standardized treadmill performance test using myometry. This study analyzes how a maximal training session influences muscle and tendon stiffness measured by MyotonPro. Thus, data for the AT and SM of professional female athletes with a comparable training load were measured before and compared to the data after a treadmill test.

As Achilles tendinopathy shows a high prevalence in professional athletes [27], better preventive programs are crucial for reducing acute and chronic injuries. As MyotonPro is a handheld, easy-access tool for collecting quantitative data on musculoskeletal tissue, data could be collected more regularly, thereby enhancing preventive programs for female professional athletes.

The second aim of this study is to examine potential differences in stiffness between different types of sports and determine whether these can be measured by myometry. The study will assess the muscle and tendon stiffness of the soleus muscle and Achilles tendon. Given that different sports exhibit highly varied locomotion patterns, it is necessary to establish baseline values for professional female athletes. These baseline values may provide better insights into the development of tendinopathy by regular measurements of the muscle–tendon unit. By examining these parameters, this study aims to provide a deeper understanding of relevant biomechanical properties across different types of sports. This may enhance the comprehension of sport-specific performance and injury mechanisms by analyzing quantitative myometry data. The novelty of this study is its focus on myometry of the AT and SM in professional female athletes, as previous studies predominantly analyzed male athletes. The findings of this study may offer insights into gender- and sport-specific differences in tendon and muscle stiffness, which is essential for improving musculoskeletal prevention.

## 2. Methods

### 2.1. Study Design

A quasi-experimental design was employed to investigate stiffness changes in the AT and SM before and after a standardized treadmill test, as measured by myometry. Due to the study design, no randomization was performed. Twenty-four female athletes (volleyball and handball) underwent a standardized exercise test on a treadmill as part of the annual basic physical examination. Furthermore, this study analyzed differences in the type of sport (volleyball *n* = 13, handball *n* = 11; hammer throw *n* = 9; swimmers *n* = 5) by using myometry. Musculoskeletal tissue was examined to gain insight into the relationship between myometry and the type of sports, as well as the athletic performance in female professional athletes. The inclusion criteria were (I) healthy female professional athletes (>10 h training per week), (II) without any acute (>6 months) musculoskeletal, rheumatic or vascular comorbidities and no previous injuries of the Achilles tendon or soleus muscle, (III) who provided written informed consent to participate in the study.

The athletes’ baseline characteristics were recorded on the examination day. On the day of the measurements and treadmill exercise test, no training was performed before the exercise test or the measurements. The running protocol was conducted on a treadmill (T170 h/p/cosmos) with a 1% incline. The treadmill velocity began at 6 km/h and was increased by 2 km/h every 3 minutes. The protocol lasted 18 to 21 min until individual exhaustion. The heart rate was continuously measured via mobile electrocardiogram, while the blood pressure and blood lactate were measured every three minutes. The lactate threshold and individual anaerobic threshold were determined after measurement. The maximal oxygen consumption (VO_2_peak) was calculated by the Ergonizer software (Version 5, Freiburg, Germany). The blood lactate was measured using a C-Line Biosen analyzer by EKF Diagnostic (Barleben, Germany).

### 2.2. Myometry Examination Protocol

A standardized protocol was applied for all the myometry measurements of the female athletes. The pre- and post-exercise measurements were performed on the same day. For the assessment of the Achilles tendon, the mid-portion stiffness was measured, which was identified by using ultrasound in advance. The participants were examined in a prone position with both feet hanging in a relaxed position over the examination couch. The leg position was maintained for the measurement of the soleus muscle (SM) insertion, which was detected by ultrasound and subsequently marked. The measuring points were initially marked to ensure the exact measuring points were used before and after the standardized running test.

### 2.3. Statistical Analysis

The mean of two measurements is recommended in the literature for robust results [7] and was performed in this study. For 2 measurement points in 38 female athletes, including post-measurements for 24 athletes, a total of 248 myometry measurements were performed. Statistical analysis for systematic deviation was conducted. The measurements were examined for a normal distribution using the Kolmogorov–Smirnov test. The student *t*-test was used for further analysis. The paired *t*-test was used to investigate the pre- and post-exercise subgroup analysis. The unpaired *t*-test was performed to examine differences between different types of sports. A significance level of *p* < 0.05 was determined and all the statistical analyses was performed using SPSS software (IBM Corp., released 2019. IBM SPSS Statistics for Windows, Version 26.0. Armonk, NY, USA: IBM Corp.). Microsoft Excel software (Microsoft Excel, released 2019, Redmond, WA, USA) was used for the boxplot visualization.

Descriptive statistical analyses of all the athletes and subgroups were examined and analyses of the pre- and post-exercise measurements were conducted. Furthermore, subgroup analysis of 38 female athletes was performed for different types of sports. Pearson correlation was conducted using SPSS to examine the body composition of all the female athletes. It was used to calculate the relationship between the AT and SM stiffness and the lactate parameters and VO_2_peak. The significance of the correlation was determined by calculating the *p*-value.

## 3. Results

### 3.1. Athletes’ Characteristics

The mean age of all 38 athletes was 20.61 years and the mean BMI was 23.49 ± 2.95 kg/m^2^. Table 1 presents the descriptive analysis and the mean values of dynamic stiffness (N/m) measured by myometry, with the minimum and maximum ranges. The mean values of stiffness (N/m) measured by myometry, with the minimum and maximum ranges, were 387 (297.5–535.5) for the SM and 650.71 (860.5–411.5) for the AT. Seven athletes reported ACL ruptures in the past, and one of these seven athletes reported ACL ruptures on both sides. One athlete reported patellar tendon rupture. None stated Achilles tendon rupture, pain, swelling or deficits in ankle movement during running or jumping in the past six months. Muscle pain or soreness of the lower limbs was not reported. One athlete took L-Thyroxin as a treatment for hypothyroidism. One athlete took a progesterone contraceptive pill and one athlete a combined contraceptive pill. No other medications were reported.

All the athletes (volleyball *n* = 13, handball *n* = 11; hammer throw *n* = 9; swimmers *n* = 5) conducted 7–10 sport-specific training sessions, with additional strength and stability training sessions.

For further examination, a descriptive analysis was conducted for the subgroup (*n* = 24) that performed a standardized treadmill test to examine the changes in AT and SM stiffness resulting from acute exercise using myometry. Besides the descriptive parameters, metabolic and speed parameters (maximal speed in km/h, speed at the individual anaerobic threshold—speed at IAT in km/h) were collected during the lactate threshold test. The relative VO_2_peak (ml/kg/bodyweight) was calculated by the software to allow a comparison of physical fitness in the exercise group.

### 3.2. Results of Subgroup Analysis before and after a Standardized Treadmill Test

The exercise group included 13 professional volleyball athletes and 11 professional handball athletes. There was no significant difference in the stiffness of the AT (left) and SM (left and right) between the volleyball and handball athletes. The handball athletes exhibited a significantly softer AT on the right side before the exercise test compared to the volleyball players. The handball athletes were significantly shorter; however, the BMI was comparable. The handball and volleyball players had comparable lactate maximum values and comparable lactate values at the IAT. The calculated relative VO_2_peak was significantly higher in the handball players (*p* = 0.007; HB: 48.75 vs. VB: 45.49 mL/min/kg/bodyweight). Furthermore, the maximum running speed was significantly higher in the handball players (Table 1).

A significant negative correlation of −0.552 (*p* < 0.01) was found between the maximum lactate value and the measured stiffness of the soleus muscle. There was also a negative correlation between the maximum lactate value and the stiffness of the Achilles tendon, but without sufficient significance. There was no significant correlation between the relative VO_2_peak and the stiffness of the AT and SM.

As expected, the running speed at the IAT correlated significantly (Pearson correlation 0.702; *p* < 0.01) with the relative VO_2_peak.

### 3.3. Results of Subgroup Analysis for Type of Sport

The hammer throwers (*n* = 9) were significantly heavier and shorter compared to the swimmers (*n* = 5). There were no significant differences in age, BMI and height. The handball players had a significantly higher BMI (*p* = 0.044) compared to the swimmers. Differences between the handball and volleyball athletes are displayed in Table 1.

**Figure 1:**
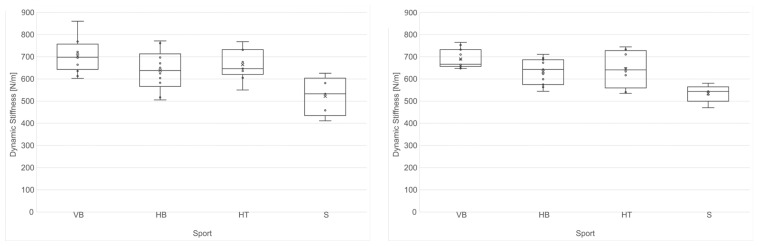
Boxplots of the stiffness of the left Achilles tendon left (**left**) and the right AT (**right**) for different sports in all 38 professional female athletes (VB: volleyball *n* = 13; HB: handball *n* = 11; HT: hammer throw *n* = 9; S: swimming *n* = 5).

**Figure 2:**
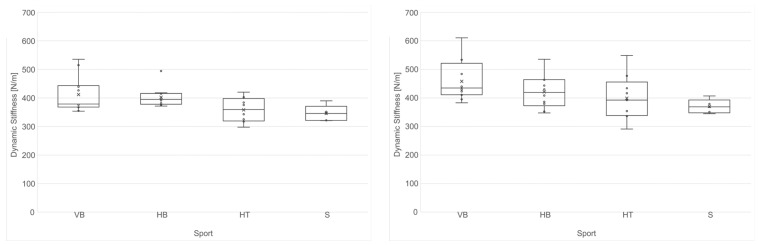
Boxplots of the stiffness of the left soleus muscle (**left**) and the right soleus muscle (**right**) for different sports in all 38 professional female athletes (VB: volleyball *n* = 13; HB: handball *n* = 11; HT: hammer throw *n* = 9; S: swimming *n* = 5).

The swimmers (522.10 N/m) had significantly softer Achilles tendons on both sides compared to the volleyball (703.71 N/m), handball (638.60 N/m) and hammer throw (664.94 N/m) athletes, while the AT stiffness did not differ between the handball, volleyball and hammer throw athletes (Figure 1). The soleus muscles showed significantly lower stiffness in the swimming athletes (345.90 N/m) compared to the handball (421.35 N/m) and volleyball (458.25 N/m) athletes. The SM stiffness did not differ between the handball, volleyball and hammer throw athletes (Figure 2).

## 4. Discussion

The type of sport is an important parameter for applying myometry in professional female athletes. The results of this study demonstrated that myometry can detect differences in stiffness in the Achilles tendon and soleus muscle in 38 professional female athletes across different sports. Furthermore, there were no differences in the Achilles tendon and soleus muscle stiffness before and after an acute exercise session. Myometry can be performed on the same day of an acute training session in healthy professional athletes. Myometry is easy to apply and can be used by trainers and physiotherapists. Easy-to-learn and point-of-care devices have considerable advantages for accessible preventive applications for monitoring muscles and tendons. The results of this prospective study are particularly important for coaches, physiotherapists, sports scientists and sports medicine physicians, especially for monitoring tendons and muscles in different sports and adapting training plans during different training phases.

### 4.1. Myometry Measurements before and after Acute Standardized Exercise

Acute running and sprinting until exhaustion challenge the muscle tendon unit, as a stiff Achilles tendon can store energy, potentially enhancing muscle–tendon efficacy [20]. Since the MyotonPro is a hand-held tool that can be used by most physiotherapists or coaches, it is necessary to examine how a single training session impacts the stiffness of the tendons and muscles. This can provide insights into whether MyotonPro can be used on training days or directly after an active warm-up. The competitive athletes were comparable in age and BMI. The handball players were significantly shorter but still had a comparable BMI. The measurements of dynamic stiffness (N/m) using myometry showed no significant differences before and after the acute running load. The MyotonPro could not measure differences or significant changes in the AT and SM dynamic stiffness before and after a running load in healthy professional athletes in this study. As the measurements of dynamic stiffness (N/m) using myometry showed no significant differences before and after the acute running load, coaches or physiotherapists can be advised to apply myometry on training days.

However, Doppler flow ultrasound, as another imaging technique, was able to detect differences in the blood flow in healthy and tendinopathic AT after a running exercise [28]. Athletes with Achilles tendinopathy showed a higher blood flow in the AT for significantly longer compared to the healthy participants. Furthermore, the blood flow in healthy ATs normalized after 30 min of rest. In the examinations after 30 min, no differences were observed. Thus, an acute treadmill exercise test may change the tendon perfusion in healthy athletes in the first thirty minutes, which does not have an influence on tendon stiffness. It can be concluded that myometry can be performed on training days with acute stress or active warm-ups.

Pozarowszczyk et al. showed a significant increase in the dynamic stiffness of the Achilles tendon, measured by MyotonPro, after an acute strength test [23]. The working group also investigated Achilles tendon stiffness before and after several karate fights, showing a significant increase in AT stiffness in the dominant leg [24] but not in the non-dominant leg. The cumulative increased stress time with multiple maximum loads on a competition day in the study by Pozarowszczyk et al. may explain the differences [24]. Due to the incremental treadmill test in our study, only a short anaerobic phase of approximately three to six minutes was reached. However, the examined athletes in Pozarowszczyk et al.’s study performed multiple maximal exertions with potential high-impacts in several karate fights on the same day [24]. Furthermore, physical demands play an essential role in muscle and tendon stiffness. The contractile properties of musculoskeletal tissue can be affected by the type of physical activity, the intensity and the duration [29]. High-intensity training or competition sessions with highly repetitive sport-specific movements can lead to early muscle adaptions, resulting in myometry changes that might also be gender-specific. Also, sport-specific muscle activation patterns in karate fights and different force production might lead to different results in the study by Pozarowszczyk et al. [24].

Differences in tendon and muscle stiffness can also be influenced in different training demands [30]. Regular resistance training showed higher muscle and tendon stiffness in women in comparison to a non-trained control group. In this examination, a valuable prediction of muscle function was shown by myometry and the authors highlighted the utility of myometry in sports [30]. Gender-specific differences in muscle activation patterns and biomechanical variations due to hormonal differences also need to be considered, as most studies on myometry predominantly examined male athletes [21,22,24]. Further research is necessary in this area.

Therefore, an application on competition days with maximal musculoskeletal stress should initially be used cautiously for preventive monitoring. Further research is needed to evaluate whether myometry should be performed during competition days to collect stiffness values as part of a prevention program, especially in case of maximal exertion. Further studies with larger populations of professional athletes are necessary to examine the Achilles tendon over several seasons, pre-season, mid-season and post-season, to understand tendon development during different training phases, considering gender-specific factors.

A further investigation of the group differences between the athletes who performed the treadmill exercise test revealed significant differences in the maximal running speed and calculated relative VO_2_peak. The examined handball players were fitter (with significantly higher relative VO_2_peak compared to the volleyball athletes) and exhibited a higher maximum running speed and softer AT on the right side. This may be due to the higher jumping load and rate of the volleyball players [8]. There was no significant correlation between the VO_2_peak and AT or SM stiffness in professional handball and volleyball athletes. Further research is required to determine if high VO_2_peak values are associated with stiffer musculoskeletal tissue, as higher Achilles tendon stiffness is associated with a better economic performance, e.g., in jumping and running [31].

### 4.2. Myometry and Type of Sport

Athletic performance results from an optimal muscle–tendon interaction [20]. To understand this interaction more precisely, it is necessary to examine the stiffness of different sports and different locomotion patterns. It is well known that regular training does have an impact on the elastic and mechanical tissue of muscles and tendons. Several studies revealed higher muscle and tendon stiffness in athletes compared to inactive individuals [32,33]. It is less understood how different locomotion patterns influence muscle and tendon stiffness, and whether these changes can be measured by myometry. Especially in sports with high vertical forces like jumping and sprinting, or those with high static contraction like hammer throw, muscles and tendons are exposed to a multiple of the individual’s bodyweight [34]. Due to repetitive training, tissue adaptions such as an increased tendon cross-sectional area and higher values of Young’s modulus can be observed [35,36]. Regular examinations with ultrasound and shear wave elastography are often still reserved for male athletes with better medical infrastructure. Dynamic stiffness is an important parameter for understanding regular training, including tissue adaption through repeated microtrauma, and for gaining insights into athletes’ tissue physiology. As an easy-to-use, point-of-care tool, myometry can address this diagnostic gap for female athletes.

This study provides crucial insights into the physiology and stiffness in different types of sports by examining professional female athletes with comparable training loads (>10 h/week) using myometry. Female swimmers showed significantly softer AT and SM compared to female athletes in handball, volleyball and hammer throw. The lower stiffness values of the AT and SM of female swimmers are particularly notable. This is likely due to the fact that swimmers rarely expose their legs to impact loading. Therefore, they also do not require high stiffness in the AT and SM. These findings are similar to the results of Kongsgaard et al. [31]. In that study, professional runners, volleyball players and kayak athletes were examined and the sport-specific adaption of the AT was shown. The kayak athletes had a significantly smaller cross-sectional area compared to the endurance running athletes and volleyball players. The kayak athletes and volleyball players also exhibited significantly higher AT stress while performing maximal plantar flexion compared to the running athletes [31]. Our results confirm that repetitive high-impact training leads to increased AT and SM stiffness. Different locomotion patterns with high force production and high impact on tendon and muscle tissue lead to an increase in muscle and tendon tissue. Genetic factors and individual training and injury history should also be considered in further studies.

Low stiffness should not present a health problem for swimming athletes. However, caution and monitoring of musculoskeletal tissue are needed for injury prevention during the transition from swimming to cycling and running in triathlon. The high prevalence of Achilles tendinopathy in running and triathlon could be addressed with regular tendon monitoring and strength training [23,27] to prevent chronic injuries. Due to the measurable differences between different types of sports, myometry could be helpful in competitive sports as a point-of-care device to detect significant differences in stiffness and possible injuries at an early stage. Examinations have shown the potential of myometry to detect tendinopathies [37]. Further studies are needed. In case of suspected injury, further imagining examinations, including the use of ultrasound, SWE-US and power Doppler-US should also be performed in order to collect qualitative parameters in addition to quantitative measurements.

The Achilles tendons of elite male soccer players were examined by myometry in comparison to healthy controls [38]. No differences in stiffness were found between the groups for the AT, but differences were evident for the patellar tendon [38]. Finnamore et al. detected significantly lower Achilles tendon stiffness in recreational runners with tendinopathy compared to healthy controls [37]. Ultrasound and shear wave elastography already allow qualitative and quantitative diagnosis of tendinopathy of the AT [39,40], showing lower tendon stiffness in cases of tendinopathy. Handheld myometry can be performed with a point-of-care device to detect early stiffness differences on the sidelines during training or a competition. Also, for inexperienced examiners, myometry shows good intra-rater reliability and can be easily applied by physiotherapists or non-medical staff [7]. This easily accessible application allows regular measurements of the sport-specific exposed muscles and tendons as part of a prevention program and could add important data for training adaptations in the pre-, post-season or rehabilitation phases. Future work will examine possible preventive programs for professional female athletes including myometry and SWE.

### 4.3. Myometry and Future Aspects

The professional female athletes examined in this study are at a high risk of acute and chronic tendinopathy due to the high impact locomotion patterns, especially in volleyball and handball. There is a lack of studies on injury prevention in women in competitive sport, although there is a significantly higher prevalence of muscular system injuries in women compared to men [41]. Tendinopathies exhibit gender-specific responses to different types of treatment. Data are often extrapolated from men to women due to a lack of studies [2,42]. However, since studies on prepubertal female athletes show no differences in the laxity of musculoskeletal tissue [43], and postpubertal data show clear differences [44], prevention strategies for female competitive athletes need to be established. In the early stages of tendinopathies, B-mode ultrasound and shear wave elastography can be helpful in detecting early qualitative and quantitative changes in muscle and tendon tissue [19,39]. Young female competitive athletes in particular show poorer neuromuscular control, with a higher prevalence of injury [45]. Myometry allows an easy-access and regular examination of sport-specific muscles and tendons, which can be helpful in detecting early differences in stiffness and the onset of a musculoskeletal injury such as tendinopathy [37]. Regular measurements could be valuable for athletes, coaches and physiotherapists, as quantitative data on stiffness measured by myometry is an additional parameter alongside manual tissue analysis and strength tests. Manual tissue analysis by physiotherapists and clinicians is also highly individual and prone to error. As there is still a lack of established predictive algorithms for sport-specific musculoskeletal injuries [46,47], a quantitative parameter could help to better understand the stiffness differences in different types of sports. Myometry might help to establish regular stiffness measurements in professional female athletes.

### 4.4. Limitations

A limitation of this study is the small number of cases in the subgroup analyses by sport (swimmers *n* = 5; VB = 13, HB = 11; HW = 9), although this is comparable to studies in competitive sports. The missing randomization needs to be considered. Comparable training units and loads in competitive sports represent a major hurdle, which is why some female athletes had to be excluded in advance due to insufficient training units.

A further limitation is the sole investigation of passive stiffness. The stiffness of active contraction was not investigated in this study. This should be addressed in further studies, as higher active stiffness was found in professional female netballers compared to recreational netball players [25]. Furthermore, the tendon length and cross-sectional area of the tendon were not measured. The tendon length is an important parameter, which can change with a higher training volume. A sport-specific decrease in the stiffness of a tendon may be a result of repetitive microtrauma. Morgan et al. hypothesized that this might lead to a change in collagen fibers and increase the risk of a tendon rupture [20].

Due to the integration of myometry measurement into the basic physical examination, it was not possible to ensure the exact measurement time directly after the running exercise. Further studies should include several measurement times (e.g., 5 min, 30 min, 60 min).

## 5. Conclusions

Myometry can be performed on the same day as an acute training session in healthy female professional volleyball and handball athletes. Female swimmers have significant lower AT and SM stiffness compared to female handball, volleyball and hammer throw athletes. These results show that the stiffness differences in the AT and SM can be assessed by myometry.

## Figures and Tables

**Table 1:** Baseline characteristics and stiffness values (N/m) of the Achilles tendon and soleus muscle before and after a standardized treadmill test for exercise subgroup (*n* = 24) (handball *n* = 11 and volleyball *n* = 13).

Variable	Exercise Subgroup (*n* = 24)	Volleyball/VB (*n* = 13)	Handball/HB (*n* = 11)	*p*-Value VB vs. HB
Age [years]	22.08 ± 3.11	21.38 ± 3.10	22.91 ± 3.05	0.239
Height [m]	1.79 ± 0.08	1.84 ± 0.07	1.73 ± 0.06	0.000
BMI [kg/m^2^]	22.51 ± 1.95	21.76 ± 1.28	23.41 ± 2.27	0.051
Max. speed [km/h]	14.87 ± 0.98	14.44 ± 0.87	15.39 ± 0.87	0.015
Max. lactate [mmol/L]	9.22 ± 2.08	8.50 ± 2.05	10.15 ± 2.01	0.158
Pre exercise				
AT				
Left	680.65 ± 82.54	703.71 ± 72.77	638.60 ± 90.86	0.085
Right	659.93 ± 59.01	690.05 ± 43.42	632.86 ± 56.67	0.016
SM				
Left	402.55 ± 45.73	458.25 ± 68.91	421.35 ± 58.44	0.675
Right	441.48 ± 65.61	411.75 ± 61.62	402.75 ± 36.03	0.189
Post exercise				
AT				
Left	668.88 ± 94.78	657.50 ± 110.27	654.750 ± 105.81	0.953
Right	640.28 ± 69.69	666.67 ± 58.93	618.35 ± 75.87	0.119
SM				
Left	386.48 ± 57.59	381.96 ± 63.27	392.36 ± 54.65	0.677
Right	443.05 ± 72.78	442.08 ± 55.32	435.15 ± 95.57	0.841
Delta Pre vs. Post				*p*-value Pre vs. Post
AT				
Left	11.77			0.427
Right	19.65			0.120
SM				
Left	16.07			0.236
Right	1.57			0.916

There was no significant difference in mean dynamic stiffness before and after acute exercise for the Achilles tendon and soleus muscle on both sides in the exercise group (Table 1).

## Data Availability

The data presented in this study are available on request from the corresponding author. The data are not publicly available due to data privacy regulations.

## Abbreviations

AT	Achilles tendon
HB	handball
HT	hammer throw
S	swimming
SM	soleus muscle
SWE	shear wave elastography
SWV	shear wave velocity
US	ultrasound
VB	volleyball
